# Exploration of subgroups and predictors of loneliness among older adults in rural China: A latent profile analysis

**DOI:** 10.1186/s12877-024-04812-w

**Published:** 2024-02-27

**Authors:** Yuecong Wang, Shasha Li, Xiaoyue Zou, Yingyuan Ni, Lijun Xu, Shufang Liao, Lijun Cao, Jianyi Bao, Yue Li, Yingxue Xi

**Affiliations:** 1 Department of Nursing, College of Medical Science, Huzhou University, 759 Second Ring Road, Huzhou, Zhejiang 313000 China; 2 Department of Personnel, The First People’s Hospital of Huzhou, No.158, Back Square Road, Wuxing District, Huzhou, Zhejiang 313000 China; 3 Internal Medicine-Cardiovascular Department, The First People’s Hospital of Huzhou, No.158, Back Square Road, Wuxing District, Huzhou, Zhejiang 313000 China

**Keywords:** Loneliness, Older adults, Latent profile analysis, Predictors

## Abstract

**Background:**

Loneliness is a negative emotional state that can lead to physical and mental health problems. This study’s objective was to acquire an in-depth understanding of the heterogeneity and the predictors of loneliness among older adults in rural China and provide valuable references for practical interventions.

**Methods:**

Older rural adults in China (*N* = 680) were recruited between January and April 2023. Latent profile analysis (LPA) was employed to identify subgroups of loneliness among participants. Single-factor and multinomial logistic regression analyses were conducted to investigate predictors of loneliness.

**Results:**

The loneliness of rural older adults could be divided into three subgroups: low interaction loneliness group (55.0%), moderate emotional loneliness group (31.8%), and high loneliness group (13.2%). The subgroup predictors included age, gender, religious beliefs, marital status, living alone, number of chronic diseases, and smartphone use (*P* < 0.05).

**Conclusion:**

This study identified a classification pattern for loneliness among older adults in rural areas of China, revealed the characteristics of different demographic variables in loneliness categories, and highlighted the heterogeneity of loneliness in this population. It serves as a theoretical reference for formulating intervention plans aimed at addressing various loneliness categories for local rural older adults.

**Clinical trial registration:**

ChiCTR2300071591.

## Background

The aging of the population has become a global problem. According to the seventh national population census conducted by the National Bureau of Statistics of China in 2020 [1], the number of people aged 60 or above in China reached 264 million, among which the rural elderly population accounted for 135 million, representing more than 50% of the total elderly population. The increasing number of rural elderly populations has raised concerns about a series of mental health problems. Studies have shown that due to the relatively limited living environment, restricted access to medical resources and cultural education, insufficient social support, and the working status of young people in urban areas, rural older adults experience intensified emotional and social loneliness caused by living alone [2]. According to a report in the Lancet, loneliness has become a serious public health issue [3]. Therefore, in-depth analysis of loneliness is key to solving the mental health problems of rural older adults.

Loneliness is defined as an unpleasant subjective experience and a negative emotional state when an individual lacks satisfactory interpersonal relationships and when there is a gap between the ideal level of social interaction and reality [4]. Studies have shown that loneliness can lead to increased levels of stress hormones such as cortisol, which can increase the risk of cardiovascular disease and other diseases [5–9]. In addition, loneliness is associated with physiological problems such as sleep issues, decreased immune function, and increased inflammation [10–12]. A national longitudinal study found that approximately 30.6% of older women and 22.9% of older men in China perceive loneliness as moderate or higher [13]. Research has shown that rural older adults have higher levels of loneliness than urban older adults, and the resulting problems of anxiety, depression, and cognitive and social dysfunction are more severe [14, 15]. Reports indicate that the burden of physical and mental illness caused by loneliness among rural older adults far exceeds the government’s investment in social medicine and health services resources [16]. Therefore, an in-depth analysis of the current state of loneliness among rural older adults will be critical for minimizing the risk of physical and mental illness among them.

The connotation of loneliness has multi-layered and multi-dimensional characteristics. Weiss distinguished between emotional loneliness and social loneliness [17]. Emotional loneliness primarily involves an individual’s sense of intimacy deprivation, occurring when their dependency and intimacy need to go unmet. Social loneliness centers around deficiencies in social relationships, manifesting when an individual perceives their social interactions as unfulfilled [17]. Gerson categorizes loneliness into state loneliness and trait loneliness, providing a different perspective for categorizing research [18]. Ditommaso’s categorization is more specific and divides loneliness into social loneliness, familial loneliness, and romantic loneliness, a classification that contributes to a more in-depth understanding of loneliness in different life domains [19]. Considering the significance of elderly individuals in loneliness research, the Loneliness Scale for Older Adults (LSOA) developed by Bandari includes five factors [20]. Compared to other instruments, the items in LSOA are suitable for monitoring the loneliness of older adults, providing more comprehensive assessment instruments for category studies.

Wu et al. utilized a US database to track 5,442 older adults, identifying latent categories of loneliness, including the low loneliness group, social loneliness group, emotional loneliness group, and high loneliness group [21]. Hsu et al. employing cluster analysis techniques, systematically examined the intrinsic structure of loneliness among older adults in the Taipei community, categorizing them into five distinct clusters [22]. In the UK, Smith & Victor subdivided the older adult community into six distinct subgroups based on the characteristics of loneliness through latent category analysis [23]. Additionally, Ermer employed growth-mixture modeling to provide insights into the shared loneliness trajectories of older couples and categorized them into three classes accordingly [24]. However, given the potential variations in performance characteristics among different groups and the heterogeneous differences in variables among individuals, along with considerations for cultural differences among various countries or regions, the applicability of what categorization to the rural older adult in China is yet to be confirmed. Moreover, there is also a lack of a “person-centered” approach to exploring the different subgroups of loneliness among rural older adults in China. Therefore, the latent profile analysis (LPA) technique provides a new perspective for identifying the profile characteristics of loneliness among rural older adults in China.

As the trend of population aging intensifies, the problem of loneliness faced by older adults in rural China is also increasing. Given the complexity and heterogeneity of loneliness, we need to address loneliness through individualized approaches. Therefore, this study aims (i) to use LPA to explore the heterogeneous characteristics of multidimensional loneliness in rural Chinese older adults, and (ii) to analyze the predictors of each latent profile and the differences in predictors among the profiles, to better address the various challenges posed by loneliness and develop targeted interventions.

## Methods

### Study design and setting

A multistage stratified whole-group random sampling method was used in this study. From January to April 2023, with Huzhou City as the total sampling pool; ten township streets were selected using the random number table method. Next, six villages were selected from each of the ten township streets sampled. Finally, 10–15 older adults aged 60 years and older were randomly selected from each village. All participants were informed of the content and purpose of this study and the benefits of participating in this project before implementation, and participants signed an informed consent form.

The living characteristic of rural older adults of Huzhou is based on home-based care, supported by communities, and supplemented by institutions. Influenced by traditional rural culture, rural older adults mainly live at home. The establishment of smart communities has led to a dependence on community activities and networked information for health promotion among rural older adults. With the expansion of coverage from hospitals, social welfare organizations, and government pension schemes, the health maintenance of rural older adults is gradually being supported by a diverse system of safeguards. This study will provide theoretical support for the formulation of policy measures targeting rural older adults with similar characteristics.

### Participants

In this study, according to the practitioner’s guide for potential category analysis published by Sinha [25], LPA is considered a suitable method for “large samples” and it is recommended that the required sample size should be greater than 500. Considering the response rate and missing rate of the questionnaire, a preliminary estimate of 40% additional respondents was calculated, and the required sample size was *n* = 500 × (1 + 40%) = 700 cases. Therefore, 700 questionnaires were eventually distributed for this study.

The inclusion criteria were as follows: (i) age ≥ 60 years; (ii) rural permanent residents (time ≥ 6 months); and (iii) clear consciousness and ability to communicate efficiently. The exclusion criteria were as follows: (i) suffering from serious acute and chronic diseases, such as severe heart failure, renal failure, liver disease, and malignancy; (ii) cognitive dysfunction assessed by the simple mental state examination (MMSE) assessment for cognitive dysfunction. First, the assessment was carried out by trained researchers. These assessors had the necessary professional knowledge and skills to use the MMSE assessment tool correctly. Second, a suitable assessment site was selected in rural areas, such as a township hospital or an activity center for the elderly. Then, the assessors used the MMSE questionnaire to conduct the assessment. They asked the respondents questions one by one and recorded their answers. Depending on the education level of the respondents, different scoring criteria were used for the assessment (MMSE test: illiterate ≤ 19, elementary school ≤ 22, junior high school and more < 26 as cognitive dysfunction [26]); and (iii) deaf, blind, aphasic or unable to communicate normally due to other physical diseases.

### Measures

The assessment instrument is a two-part structured questionnaire consisting of the general information questionnaire and the LSOA.

#### General information questionnaire

The researchers created the questionnaire, which included age, gender, religion, education level, marital status, living alone, economic status, smartphone use, number of chronic diseases, and self-assessed health status.

#### LSOA

The LSOA was developed by Bandari in 2021 to measure multidimensional levels of loneliness in older adults aged 60 years and older [20]. The scale consists of 5 factors and 29 items. It includes decreased social competence (Item 1 to Item 7), disappointment and uselessness (Item 8 to Item 14), psychological suffering (Item 15 to Item 22), experiencing periods of loneliness (Item 23 to Item 26), and ineffective interactions (Item 27 to Item 29). Scoring was performed using a 5-point Likert scale ranging from 1 (strongly disagree) to 5 (strongly agree), with a total score range of 29 to 145. The Chinese LSOA version has undergone cross-cultural adaptation and validation and demonstrated good reliability and validity [27]. In this study, the total scale and subscales of Cronbach’s alphas were 0.924 and 0.840 ~ 0.913.

### Data analysis

First, statistical analysis of the data was performed using SPSS 25.0 (version 25.0, IBM Corp., Armonk, NY) and MPLUS 8.3 (version 8.3, macOS). Descriptive statistical analyses were performed for general information, with measures expressed as the mean ± standard deviation and counts expressed as the number of cases and percentages.

Second, the 29 items of the LSOA were used as exogenous variables, LPA was used to determine the potential profiles of loneliness among rural older adults, and the optimal latent profile model was judged based on the data results. The LPA consisted of the following steps: (i) First, it was assumed that there was only one profile, i.e., the exogenous variables were sufficiently independent. (ii) The number of profiles was gradually increased and the parameter values of each model were calculated. (iii) The model fit according to the main evaluation indices of the model were evaluated, including Akaike’s information criterion (AIC), Bayesian information criterion (BIC), adjusted Bayesian information criterion (aBIC), entropy, Lo-Mendell-Rubin test (LMRT) and bootstrap likelihood ratio test (BLRT). Among them, smaller statistical values of AIC, BIC, and aBIC indicated better model fit [28]. Entropy is an index used to assess the accuracy of profile delineation and takes values between 0 and 1. When the entropy value is approximately equal to 0.8, the accuracy of the model classification exceeds 90%, and a higher entropy value indicates a higher accuracy of the classification [29]. LMRT and BLRT are used to compare the fit differences of the models, and if the p values corresponding to LMRT and BLRT reach a significant level, the k-profile models are thereby shown to be better than the k-1 profile models. When the profile models preferred by each evaluation index are inconsistent, the results of each index and the principle of model interpretability are measured together so that the best model is finally selected.

Then, differences in general information across latent profiles of multidimensional loneliness among rural older adults were analyzed and compared using chi-square tests, Fisher’s exact test, Monte Carlo simulation tests, and multinomial logistic regression. The significance test level was set at α = 0.05, and a two-sided test of *P* < 0.05 was taken as indicative of a statistically significant difference.

## Results

### Sample characteristics

This study used a total of 700 questionnaires, and 680 of them were successfully returned, yielding a valid return rate of 97.1%. The participants ranged in age from 60 to 91, with a mean age of 70.14 ± 7.28 years. Of them, 54.7% were female and 45.3% were male. Table 1 displays more traits.

**Table 1:** Demographic characteristics of participants

Characteristics	N	%/M ± SD
**Age**		
60–69	351	51.6
70–79	249	36.6
≥ 80	80	11.8
**Gender**		
Male	308	45.3
Female	372	54.7
**Religion**		
Yes	124	18.2
No	556	81.8
**Marital status**		
Married	482	70.9
Single/divorced/widowed	198	29.1
**Education**		
Primary school and less	440	64.7
Middle school	148	21.8
Senior high school or more	92	13.5
**Self-assessment of health status**		
Poor	276	40.6
General	223	32.8
Better	181	26.6
**Living alone**		
Yes	160	23.5
No	520	76.5
**Economic status (yuan/month)**		
< 1000	420	61.8
1000–3000	189	27.8
>3000	71	10.4
**Number of chronic diseases**		
0	84	12.4
1	254	37.4
≥ 2	342	50.3
**Use smartphone**		
Yes	106	15.6
No	574	84.4
**LSOA** (score, *M ± SD)*	—	72.34 ± 20.18

### Model fit indices of LPA

The model fitting results are shown in Table 2. The results show that AIC, BIC, and aBIC gradually decrease with the increase in the number of profiles. The highest entropy value was reached in the 3-profile group, and both LMRT and BLRT were significant (*P* < 0.001). However, the entropy value starts to show a decreasing trend in the 4 profile, and the LMRT is not significant (*P* > 0.05), which indicates that the model fit of the 4 profile is not better than that of the 3 profile. Considering the classification results of the model and the interpretable practical significance, we finally chose the 3-profile model as the optimal model. To further verify the reliability of the classification results, the probabilities of correct classification of multidimensional loneliness among rural older adults in the three profiles were calculated as 99.2%, 99.2%, and 99.1%. All classification results were > 98%, which indicates the high reliability of the model results in this study [30].

**Table 2:** Fitting statistics for a latent profile model of loneliness in rural older adults

Model	Loglikelihood	AIC	BIC	aBIC	Entropy	P value		Profile probability
						LMRT	BLRT	
1-profile	-31842.132	63800.263	64062.545	63878.388	N/A	N/A	N/A	1
2-profile	-29804.233	59784.467	60182.411	59903.001	0.957	< 0.001	< 0.001	0.60/0.40
**3-profile**	**-28698.528**	**57633.055**	**58166.662**	**57791.998**	**0.979**	**< 0.001**	**< 0.001**	**0.55/0.32/0.13**
4-profile	-28150.960	56597.921	57267.191	56797.273	0.968	0.190	< 0.001	0.36/0.20/0.31/0.13
5-profile	-27750.936	55857.872	56662.804	56097.634	0.963	0.224	< 0.001	0.14/0.29/0.16/0.27/0.13

*Note*: The model solution chosen is bolded

### Description of the LPA

**Fig. 1:**
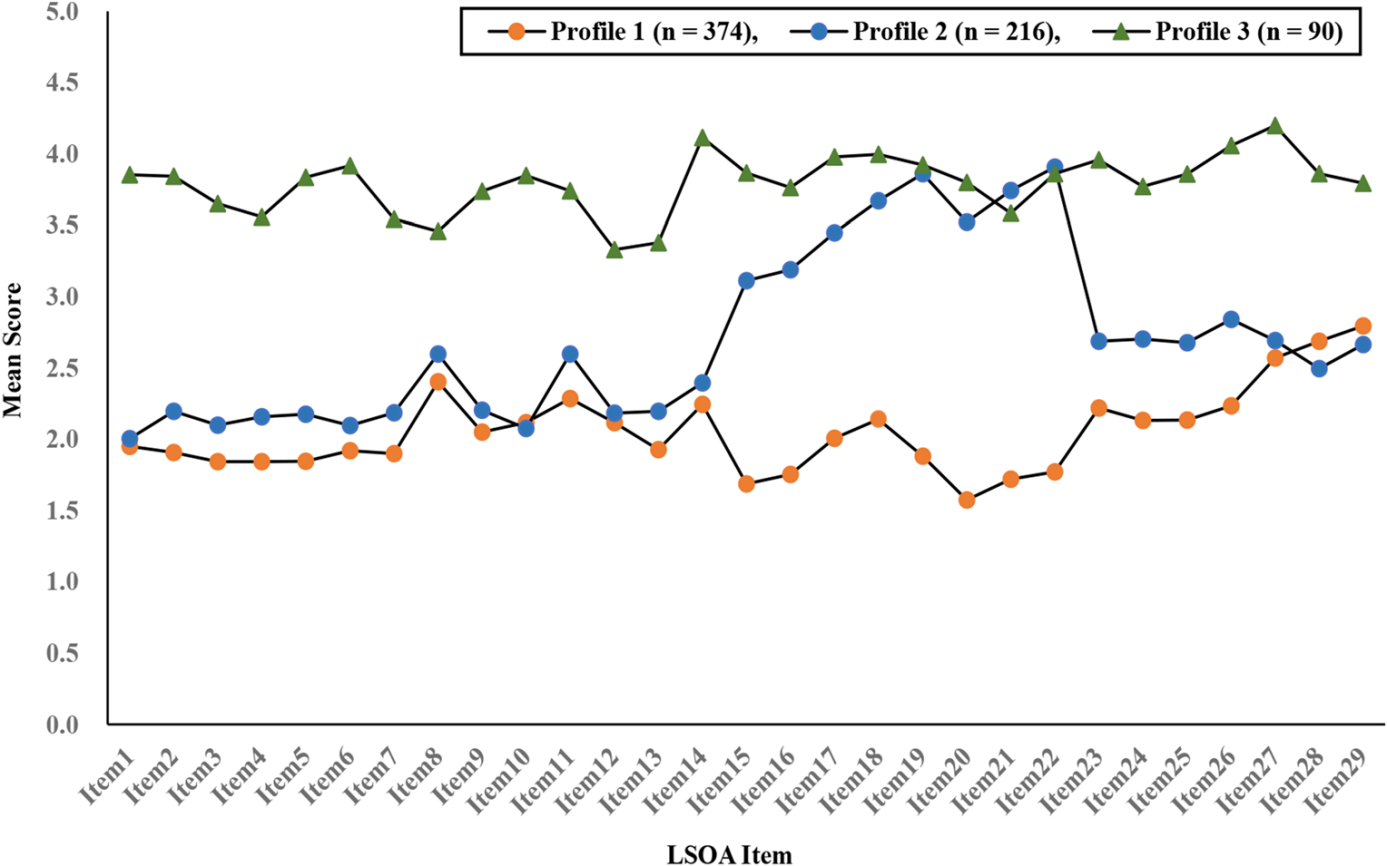
Three latent Profiles of the LSOA. *Note* LSOA consists of 5 factors and 29 items. Factor 1: decreased social competence, Item 1 to Item 7. Factor 2: disappointment and uselessness, Item 8 to Item 14. Factor 3: psychological suffering, Item 15 to Item 22. Factor 4: experiencing periods of loneliness, Item 23 to Item 26. Factor 5: ineffective interactions, Item 27 to Item 29

Figure 1 shows the subtypes of multidimensional loneliness among rural older adults (Profile 1, Profile 2, and Profile 3). Specifically, Profile 1, named the low interaction loneliness group, had a low overall loneliness score (59.75 ± 11.62) and accounted for 55.0% of the total sample; it was the most numerous subtype of the three profiles, with the highest scores on items 27 to 29 (factor 5: ineffective interactions). Profile 2, named the moderate emotional loneliness group, had a medium overall loneliness score (78.34 ± 9.55) and accounted for 31.8% of the total sample; it exhibited higher scores on items 15 to 22 (factor 3: psychological suffering). Profile 3, named the high loneliness group, exhibited overall loneliness scores that were all at a high level (110.27 ± 10.58) and accounted for 13.2% of the total sample.

### Single-factor analysis

According to the findings of the bivariate analysis, there were significant differences in the multidimensional loneliness experienced by rural older adults in terms of age, gender, religion, marital status, educational level, living alone, number of chronic illnesses, and smartphone use (*p* < 0.05), as shown in Table 3.

**Table 3:** Bivariate analysis of different latent profiles of multidimensional loneliness among rural older adults (*n* [%])

Variable	Low interaction loneliness group (*n* = 374)	Moderate emotional loneliness group (*n* = 216)	High loneliness group (*n* = 90)	X^2^	P
**Age**				149.466	**< 0.001**
60–69	249 (70.9)	98 (27.9)	4 (1.1)		
70–79	104 (41.8)	75 (30.1)	70 (28.1)		
≥ 80	21 (26.3)	43 (53.8)	16 (20.0)		
**Gender**				71.833	**< 0.001**
Male	222 (72.1)	70 (22.7)	16 (5.2)		
Female	152 (40.9)	146 (39.2)	74 (19.9)		
**Religion**				89.170	**< 0.001**
Yes	112 (90.3)	10 (8.1)	2 (1.6)		
No	262 (47.1)	206 (37.1)	88 (15.8)		
**Marital status**				36.229	**< 0.001**
Married	296 (61.4)	142 (29.5)	44 (9.1)		
Single/divorced/widowed	78 (39.4)	74 (37.4)	46 (23.2)		
**Education**				11.707	**0.020**
Primary school and less	240 (54.5)	134 (30.5)	66 (15.0)		
Middle school	72 (48.6)	58 (39.2)	18 (12.2)		
Senior high school or more	62 (67.4)	24 (26.1)	6 (6.5)		
**Self-assessment of health status**				3.292	0.510
Poor	142 (51.4)	93 (33.7)	41 (14.9)		
General	132 (59.2)	64 (28.7)	27 (12.1)		
Better	100 (55.2)	59 (32.6)	22 (12.2)		
**Living alone**				98.306	**< 0.001**
Yes	58 (36.3)	44 (27.5)	58 (36.3)		
No	316 (60.8)	172 (33.1)	32 (6.2 )		
**Economic status (yuan/month)**				8.802	0.066
< 1000	218 (51.9)	135 (32.1)	67 (16.0)		
1000–3000	113 (59.8)	61 (32.3)	15 (7.9)		
>3000	43 (60.6)	20 (28.2 )	8 (11.3)		
**Number of chronic diseases**				131.365	**< 0.001**
0	39 (92.9)	4 (4.8)	2 (2.4)		
1	178 (70.1)	58 (22.8)	18 (7.1)		
≥ 2	118 (34.5)	154 (45.0)	70 (20.5)		
**Use smartphone**				30.427	**< 0.001**
Yes	80 (75.5)	24 (22.6)	2 (1.9)		
No	294 (51.2)	192 (33.4)	88 (15.3)		

### Multinomial logistic regression analysis

The factors with *P* < 0.05 in the Single-factor analysis were selected as independent variables and included in the multinomial logistic regression for data analysis. The low interaction loneliness group was selected as the reference group, and the results showed that age, gender, religion, marital status, living alone, number of chronic diseases, and use of smartphones were the predictors of the multidimensional loneliness latent profile of rural older adults (*P* < 0.05). Table 4 displays the results.

**Table 4:** Multinomial logistic regression of multidimensional loneliness latent profiles of rural older adults

Variable	Low interaction loneliness group (Ref)						
	Moderate emotional loneliness group				High loneliness group		
	OR	95% CI	P value		OR	95% CI	P value
**Age** (Ref: 60–69 )							
≥ 80	4.102	2.111–7.970	**< 0.001**		30.463	7.991-116.133	**< 0.001**
70–79	1.641	1.040–2.591	**0.033**		33.247	10.829-102.074	**< 0.001**
**Gender** (Ref: Male)							
Female	2.104	1.378–3.211	**0.001**		5.320	2.524–11.213	**< 0.001**
**Religion** (Ref: No)							
Yes	0.186	0.089–0.390	**< 0.001**		0.079	0.016–0.379	**0.002**
**Marital status** (Ref: Married)							
Single/divorced/widowed	1.682	1.065–2.656	**0.026**		2.888	1.469–5.680	**0.002**
**Education** (Ref: Primary school and below)							
Senior high school or more	1.038	0.541–1.993	0.910		0.515	0.142–1.865	0.312
Middle school	1.651	0.990–2.751	0.055		0.647	0.273–1.531	0.321
**Living alone** (Ref: No)							
Yes	0.963	0.568–1.632	0.888		5.758	2.841–11.669	**< 0.001**
**Number of chronic diseases** (Ref: 0)							
≥ 2	15.588	5.230-46.465	**< 0.001**		23.324	3.655-148.863	**0.001**
1	4.212	1.405–12.632	**0.010**		4.713	0.715–31.051	0.107
**Use smartphone** (Ref: No)							
Yes	0.140	0.077–0.256	**< 0.001**		0.017	0.004–0.081	**< 0.001**

Moderate emotional loneliness group vs. Low interaction loneliness group: The moderate emotional loneliness group was more likely to be associated with the aged of 70–79 years (OR: 1.641, *P* = 0.033, CI: 1.040–2.591), the aged of ≥ 80 years (OR: 4.102, *P* < 0.001, CI: 2.111–7.970), being female (OR: 2.104, *P* = 0.001, CI: 1.378–3.211), being single/divorced/widowed (OR: 1.682, *P* = 0.026, CI: 1.065–2.656), with one chronic disease (OR: 4.212, *P* = 0.010, CI: 1.405–12.632) or having ≥ two chronic illnesses (OR: 15.588, *P* < 0.001, CI: 5.230-46.465). The low interaction loneliness group was more likely to be associated with having religious beliefs (OR: 0.186, *P* < 0.001, CI: 0.089–0.390) and using smartphones (OR: 0.140, *P* < 0.001, CI: 0.077–0.256).

High loneliness group vs. Low interaction loneliness group: The high loneliness group was more likely to be associated with the aged of 70–79 years (OR: 33.247, *p* < 0.001, CI: 10.829-102.074), the aged of ≥ 80 years (OR: 30.463, *p* < 0.001, CI: 7.991-116.133), being female (OR: 5.320, *P* < 0.001, CI: 2.524–11.213), being single/divorced/widowed (OR: 2.888, *P* = 0.002, CI: 1.469–5.680), living alone (OR: 5.758, *P* < 0.001, CI: 2.841–11.669), and having ≥ 2 chronic diseases (OR: 23.324, *P* = 0.001, CI: 3.655-148.863). The low interaction loneliness group was more likely to be associated with having religious beliefs (OR: 0.079, *P* = 0.002, CI: 0.016–0.379) and using smartphones (OR: 0.017, *P* < 0.001, CI: 0.004–0.081).

## Discussion

This study provides insight into the loneliness of older adults in rural areas. As far as we know, this is the first study to use LPA to explore multidimensional loneliness in rural Chinese older adults. It identifies the latent profile structure of different individuals in terms of multidimensional loneliness and related predictors. These findings provide targeted recommendations for interventions and offer new perspectives and methods for further research in this area.

In this study, a survey of 680 rural Chinese older adults using LPA revealed that the multidimensional loneliness of rural older adults could be categorized into three potential profiles, namely, a low interaction loneliness group, a moderate emotional loneliness group, and a high loneliness group, by comparing the results of model fitting, the subjects’ response characteristics on each item, and the practical implications associated with the model. The results of this categorization are consistent with the findings of Ermer’s study [24], but there is a discrepancy with other studies [22, 23]. In contrast to previous studies [31, 32], this study opted for a multidimensional loneliness scale designed for older adults, which can comprehensively reflect various categories of loneliness characteristics among rural older adults.

Notably, the percentage of each profile was > 10%, which met the minimum requirement of at least > 5% for all profiles [33]. The low interaction loneliness group accounted for approximately 55% of the total population and was the most populous group in the three profiles, with higher scores on items 27 to 29 (factor 5: ineffective interactions) and generally lower scores on the other items. The moderate emotional loneliness group accounted for approximately 31.8% of the total sample and had a moderate overall loneliness score, with higher scores on items 15 to 22 (factor 3: psychological suffering). We found that the high loneliness group accounted for approximately 13.2% of the total sample and had high levels of loneliness scores for all items. Moreover, the overall score of the high loneliness group (110.27 ± 10.58) was significantly higher than that of the moderate emotional loneliness group (78.34 ± 9.55) versus the low interaction loneliness group (59.75 ± 11.62), which further indicates better heterogeneity among subgroups.

An astonishing discovery was that these three potential profiles exhibited varying scores on the loneliness factor, reflecting distinct experiences and feelings among different rural older adults when confronting loneliness. The low interaction loneliness group primarily encounters challenges in interpersonal communication, scoring higher on the ineffective interaction factor. This may be linked to a lack of effective social support or communication barriers, making it difficult for them to engage in in-depth discussions or causing them to feel increasingly distant from friends and family. The moderate emotional loneliness group suffered greater psychological pain, possibly stemming from unmet emotional needs or insufficient emotional support. Despite having lower scores on some loneliness factors, their higher scores on the psychological suffering factor indicate underlying emotional distress and loneliness. The high loneliness group demonstrated elevated higher scores on all loneliness factors, further confirming that they face significant challenges with loneliness on multiple levels. This group of older adults not only faces difficulties in interpersonal communication but also experiences a decline in social skills and internal psychological pain. Additionally, struggling with feelings of disappointment and a sense of worthlessness, long-term loneliness can lead older adults to experience the highest levels of loneliness.

Studies have confirmed the predictive effect of age, gender, religion, marital status, living alone, number of chronic illnesses, and smartphone use on multidimensional loneliness in rural older adults.

In terms of age, those ≥ 70 years are more likely to be in the moderate emotional loneliness and high loneliness groups. Studies have shown that loneliness in older adults tends to increase with age to varying degrees and peaks at age 80 and above [34]. Loneliness in older adults can be attributed to several reasons. First, the solidification of traditional concepts leads to moderate social relational loneliness. According to traditional beliefs, older adults should stay at home and take care of their families; however, this can lead to a smaller social circle and make communication and engagement with the outside world difficult, thus triggering long-term social relational loneliness. Second, the loss of attachments (e.g., widowhood, divorce) [16] and the “empty nest” phenomenon caused by urban-rural migration can also deepen the sense of emotional loneliness among older adults. These events can cause older adults to lose important intimate relationships, exacerbating their sense of loneliness.

In terms of gender, women were more likely to be in the moderate emotional loneliness group and high loneliness group than men. This may be because older women are more willing to go out and reveal their true inner feelings to others, while older men are better at hiding their negative emotions [35]. In terms of religious beliefs, older adults with religious beliefs are more likely to belong to the low interaction loneliness group because religious beliefs give older adults spiritual support, which makes lonely and helpless rural older adults belong to the same group through religious beliefs, gaining a sense of belonging and therefore relieving their loneliness [36].

Regarding marital status, older adults who are unmarried/divorced/ widowed are more likely to be in the moderate emotional loneliness group and high loneliness group compared to those who are married. Stable marital status is essential in maintaining older adults’ mental health. Being involved in the closest relationship in the social network of older adults, those with a spouse can bring more companionship and trust to each other, while those without a partner (unmarried/divorced/widowed) can be affected by a series of negative life events in their lives, thus increasing their level of loneliness [37].

Regarding living alone, rural older adults living alone are more likely to be in the high loneliness group than those who do not live alone. This is because rural older adults living alone have the dual attributes of living alone and being old [15], making them more vulnerable in the elderly population. Additionally, this study’s results indicate that living alone is a critical factor in differentiating the moderate emotional loneliness group from the high loneliness group. The moderate emotional loneliness group primarily stems from psychological distress rather than living alone. In contrast, living alone is a significant risk factor for the high loneliness group. Lack of limited daily communication and a scarcity of social support resources exacerbate the levels of loneliness among rural older adults.

Regarding the number of chronic diseases, people with ≥ 2 chronic diseases were more likely to be in the moderate emotional loneliness group and the high loneliness group compared to those with no chronic diseases. In the present study, more than one-half of the rural older adults had ≥ 2 chronic diseases, a result higher than that of Zhang et al. [38]; the result was both physical and psychological distress, and physical functioning and quality of life were seriously affected [39–41].

Surprisingly rural older adults who use smartphones are more likely to belong to the low interaction loneliness group. Meanwhile, the intergenerational distance continues to widen, and the communication between children and parents is becoming less and less frequent, narrowing the social circles of rural older adults and making it more difficult for them to interact and communicate with the outside world [42]. In this context, using smartphones has become essential for rural older adults to maintain social connections. Compared to urban older adults, rural older adults have fewer opportunities for leisure and entertainment, and they use smartphones for recreation and social interaction (e.g., browsing TikTok), thus alleviating their internal loneliness [43]. However, the increased use of smartphones leads to some limitations in the social behaviors of rural older adults, thus contributing to the fact that they are more likely to belong to the low interaction loneliness group.

### Strengths, limitations, and future directions

Our study is not only the first to involve applying LPA to loneliness in rural Chinese older adults but is also unique in its use of a validated specificity assessment tool. However, there are still some critical limitations that require consideration. Firstly, it’s crucial to mention that our sample was limited to the Huzhou region of China, which may under-represent other populations. Therefore, in future investigations, we aim to expand our sample by including additional regions to validate our findings. Secondly, due to the use of self-report questionnaires, the research results are susceptible to the influence of memory bias. Thirdly, in our data analysis, we only investigated the relationship between demographic variables and loneliness profiles among rural older adults, without considering other potential factors and more detailed demographic classifications, such as the categorization of religious beliefs. This may result in explanations that are not sufficiently comprehensive. Finally, cross-sectional studies cannot explain the causal relationships between variables. Hence, future studies could utilize a longitudinal tracking research method to investigate the trends in loneliness among rural older adults.

## Conclusion

Overall, this study clarifies the subgroup characteristics of loneliness among rural older adults in China and highlights the connection between demographic variables and loneliness. Specifically, loneliness heterogeneity in rural Chinese older adults is categorized into three profiles: the low interaction loneliness group, moderate emotional loneliness group, and high loneliness group, offering robust evidence for the diversity of loneliness among rural older adults. Predictive factors encompass age, gender, religion, marital status, living alone, number of chronic diseases, and smartphone use. From a policy perspective, understanding the factors that influence loneliness among rural older adults can inform the development of targeted interventions aimed at strengthening the social connectedness and well-being of this population. In practice, these results guide designing interventions to meet the specific needs of individuals within each loneliness profile. Future research could further explore the effectiveness of these interventions and delve into other factors that contribute to loneliness in this population. Thus, this study provides a theoretical foundation for dealing with the issue of loneliness among older adults in rural in the future.

## Data Availability

The datasets used and analyzed in this study are available from the corresponding author upon reasonable request.

## Abbreviations

LSOA: The loneliness scale for older adults
LPA: Latent profile analysis
AIC: Akaike information criteria
BIC: Bayesian information criteria
aBIC: adjusted Bayesian information criterion
LMRT: Lo-Mendell-Rubin test
BLRT: Bootstrap likelihood ratio test
